# The Utility of Contrast-Enhanced Magnetic Resonance Imaging in Uterine Cervical Cancer: A Systematic Review

**DOI:** 10.3390/life13061368

**Published:** 2023-06-12

**Authors:** Giacomo Avesani, Alessio Perazzolo, Andrea Amerighi, Veronica Celli, Camilla Panico, Evis Sala, Benedetta Gui

**Affiliations:** 1 Department of Diagnostic Imaging, Oncological Radiotherapy and Hematology, Fondazione Policlinico Universitario “A. Gemelli” IRCCS, 00168 Rome, Italy; giacomo.avesani@policlinicogemelli.it (G.A.); veronica.celli@guest.policlinicogemelli.it (V.C.); evis.sala@policlinicogemelli.it (E.S.); benedetta.gui@policlinicogemelli.it (B.G.); 2 Department of Radiological and Hematological Sciences, Università Cattolica del Sacro Cuore, 00168 Rome, Italy; alessio.perazzolo01@icatt.it (A.P.); andrea.amerighi01@icatt.it (A.A.)

**Keywords:** cervical, cancer, MRI, CE-MRI, contrast

## Abstract

**Simple Summary:**

According to the latest ESUR guidelines, T2WI and DWI-MR sequences are fundamental for initial staging, treatment response assessment, and evaluation of recurrence in cervical cancer, while contrast-enhanced MRI (CE-MRI) remains optional; this systematic review aims to give an overview of the literature regarding CE-MRI in cervical cancer. A total of 98 papers were included. We did not find strong evidence suggesting that CE-MRI is helpful in the clinical setting for cervical cancer staging and detection of tumor recurrence. Perfusion parameters and perfusion-derived radiomics models might have a role as a prognostic and predictive biomarker but more extensive multicentric studies with robust external validation are needed to introduce it in daily clinical practice.

**Abstract:**

Correct staging of cervical cancer is essential to establish the best therapeutic procedure and prognosis for the patient. MRI is the best imaging modality for local staging and follow-up. According to the latest ESUR guidelines, T2WI and DWI-MR sequences are fundamental in these settings, and CE-MRI remains optional. This systematic review, according to the PRISMA 2020 checklist, aims to give an overview of the literature regarding the use of contrast in MRI in cervical cancer and provide more specific indications of when it may be helpful. Systematic searches on PubMed and Web Of Science (WOS) were performed, and 97 papers were included; 1 paper was added considering the references of included articles. From our literature review, it emerged that many papers about the use of contrast in cervical cancer are dated, especially about staging and detection of tumor recurrence. We did not find strong evidence suggesting that CE-MRI is helpful in any clinical setting for cervical cancer staging and detection of tumor recurrence. There is growing evidence that perfusion parameters and perfusion-derived radiomics models might have a role as prognostic and predictive biomarkers, but the lack of standardization and validation limits their use in a research setting.

## 1. Introduction

Cervical cancer is the fourth most frequently diagnosed cancer and the fourth leading cause of cancer death in women, with an estimated 604,000 cases and 342,000 deaths in 2020 worldwide. Its prevalence is strongly associated with socioeconomic status, partly due to differences in medical care and screening access. It is the second-most common cancer in low-income countries [1].

The etiology of cervical cancer is related to long-term human papillomavirus infections (HPV) [2]. Although most recent observations indicate that HPV can play an etiological role in many cancers [3], HPV infection represents the most prevalent sexually transmitted disease. In particular, it results in over 14,000,000 individuals annually and 80% of sexually active individuals in their lifetime being infected with HPV [4]. The most common histology is squamous cell carcinoma (SCC), accounting for 70–80% of all cervical cancers; non-squamous histology, such as adenocarcinoma (AC), is associated with a worse prognosis [5].

Cervical cancer staging was historically based on the Federation of Gynecology and Obstetrics (FIGO) guidelines, which were based exclusively on clinical examination until 2018 when staging criteria were revised to incorporate imaging and pathologic findings; the significant updates are represented by [6]:

- Stage I, confined to the cervix: IA (invasive carcinoma with a maximum depth of invasion ≤5 mm) and IB (invasive carcinoma with a maximum depth of invasion >5 mm and divided according to dimensions into IB1, IB2, and IB3, with invasions of ≤2 cm, >2 cm–≤4 cm and >4 cm, respectively);
- Stage II: remains unchanged with IIA (invasive carcinoma limited to the 2/3 of the vagina without parametrial invasion and divided according to dimensions into IIA1 and IIA2, with invasions of ≤4 cm and >4 cm, respectively) and IIB (invasive carcinoma limited to the 2/3 of the vagina with parametrial invasion);
- Stage III: invasion of the lower third of the vagina (IIIA), involvement of the pelvic sidewall and/or hydronephrosis or non-functional kidney (IIIB), and the presence of lymph node metastases (including micrometastases) in pelvic (IIIC1) and/or paraaortic regions (IIIC2);
- Stage IV: remains unchanged with IVA (represented by extension to the adjacent organs, including biopsy-proven invasion of the bladder or rectal mucosa) and IVB (represented by distant metastases, including lymph node metastases beyond pelvic and paraaortic regions).

Correct disease staging is essential to establish the best therapeutic procedure and prognosis for the patient. ESGO-ESTRO-ESP (European Society of Gynaecological Oncology-European Society for Radiotherapy and Oncology-European Society of Pathology) 2018 guidelines recommend surgery for early stage (IB1, IB2, IIA1) and concomitant chemoradiotherapy (CCRT) as the treatment of choice for large cervical cancer (stages IB3, IIA2) and advanced disease (≥stage IIB) [6].

MRI (magnetic resonance imaging) is the best imaging modality for local staging of cervical cancer as it has a high accuracy to assess the extent of the disease within the cervix and its extent in the vagina, the uterus, the parametrium, and the pelvis, thus impacting treatment choice and planning [7]. On the other hand, PET/CT (positron emission tomography/computed tomography) is the preferred option to assess the nodal status and distant metastasis, also for treatment planning before CCRT with curative intent. Furthermore, following chemoradiotherapy, MRI is used to assess tumor response, evaluate recurrence, and guide further treatment options. Moreover, MRI is the modality of choice to evaluate the presence of eligibility criteria for fertility-sparing (FS) treatment.

According to the latest ESUR (European Society of Urogenital Radiology) guidelines, T2-weighted imaging (T2WI) and diffusion-weighted imaging magnetic resonance (DWI-MR) sequences, ideally matched in terms of the acquisition plane, field of view, and slice thickness to allow side-by-side interpretation, are fundamental for initial staging, treatment response assessment, and evaluation of recurrence. In particular, T2WI allows one to obtain anatomical and morphological information and DWI-MR allows one to obtain information about water molecules’ motions and consequently about the cellularity of the tissue. In the same guidelines, CE-MRI (contrast-enhanced MRI) remains optional [8] and further details are not provided. However, it needs to be specified when using contrast might be helpful. Therefore, this systematic review aims to give an overview of the literature regarding the use of contrast in MRI (CE-MRI) in patients with cervical cancer and to provide more specific indications of when it may be helpful.

## 2. Materials and Methods

The Preferred Reporting Items for Systematic Review and Meta-Analyses (PRISMA) checklist was followed for our review. Registration in the international prospective register of systematic reviews (PROSPERO) was not performed.

The checklist is available in Supplementary Table S1.

### 2.1. Information Sources and Search Strategy

Systematic searches on PubMed and Web Of Science (WOS) were performed using the following advanced search keywords: “(MRI OR magnetic resonance) AND (cervical OR cervix) AND (cancer OR carcinoma OR tumor) AND (contrast OR Dynamic contrast-enhanced OR DCE)” in November 2022. The literature search was not limited by language or study type. However, the literature search was limited between 1990 and 2022.

### 2.2. Eligibility Criteria

The inclusion criteria were the following:

- Presence of CE-MRI sequences;
- Diagnosis of uterine cervical cancer.

The exclusion criteria were the following:

- Review articles;
- Non-English articles;
- Abstract texts without full paper;
- Studies about combined imaging techniques (e.g., PET-MRI).

### 2.3. Study Selection

The searches performed using the two search engines were combined, deleting the duplicate articles.

Two authors independently performed a first article selection excluding all non-eligible papers based on the presence of the keywords in their titles or abstracts. Secondary screening involved extracting the complete text, which was again reviewed by both authors, paying attention to compliance with the eligibility and exclusion criteria. A third experienced author solved any discordance in the selection process.

### 2.4. Data Extraction

From the selected papers, relevant data were extracted, in particular, the following:

- Study characteristics (publication year and design);
- Patient’s characteristics (number of patients);
- Characteristics of MRI contrast sequences;
- Objective of the study;
- Outcome measured;
- Results obtained;
- Statistical relevance of the results obtained when adequately stated.

### 2.5. Quality Assessment

Quality assessment was conducted using the Quality Assessment of Diagnostic Accuracy Studies-2 tool, which includes four domains: patient selection, index test, reference standard, and flow and timing [9]; each domain was valued in terms of risk of bias; patient selection, index test, and reference domains were assessed in terms of concerns regarding applicability. Each element was valued to detect low, high, or unclear risk. Two authors independently evaluated the methodological quality of the included articles and any disagreement was resolved by consensus.

Regarding patient selection, the presence of a consecutive or causal sample, the avoidance of case-control studies, and the possible exclusion of patients from the analysis were evaluated in terms of risk bias, and homogeneity of the study population (for example, by disease stage, age, etc.) was considered for applicability.

Regarding the index test, interpretation of the test without knowing the results of the reference standard, the use of the same MRI protocol over time, and the avoidance of the use of MRI machines with different magnetic fields were evaluated in terms of risk bias, and the direct comparison between contrast sequences and non-contrast sequences and the use of the diffusion sequences were evaluated in terms of applicability.

Reference standards based on histopathological data and independent interpretation were considered adequate in terms of risk bias and their univocality was assessed in terms of applicability.

The time interval, the homogeneity of the treatments performed on the different patients, and the inclusion of all patients enrolled in the analysis were evaluated on the flow and timing for the risk of bias.

## 3. Results

### 3.1. Study Results

**Figure 1:**
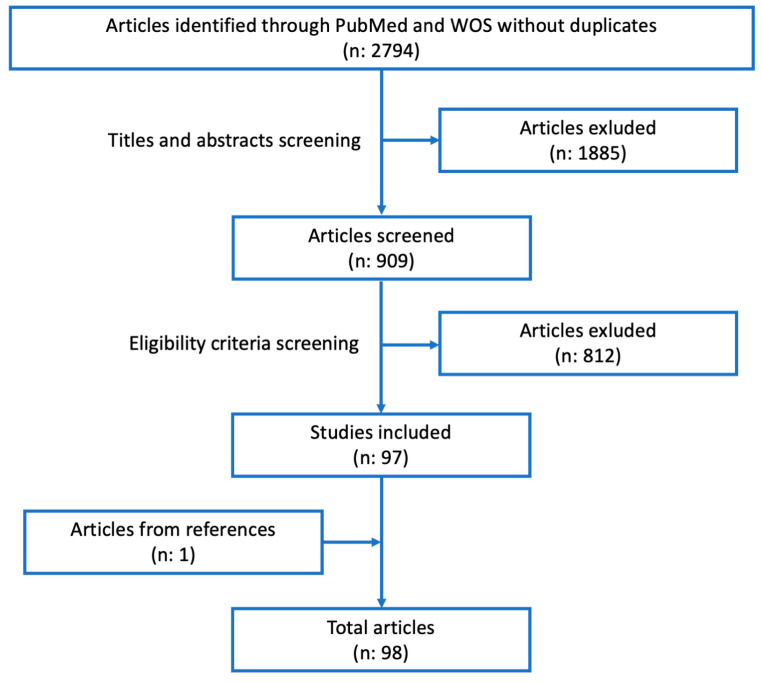
Flowchart of the study.

The initial database search provided 2794 articles after the removal of duplicates. However, during the first screening of their titles and abstracts, 1885 articles were removed because they needed to be more pertinent. Another 812 themes were removed because they did not respect eligibility or exclusion criteria. In the end, 97 papers were included. Considering the references of included articles, we found one paper that could be included, and it was added (Figure 1).

The papers had the following main subject:

- 29 on the diagnosis;
- 3 on post-treatment evaluation;
- 5 on recurrence;
- 4 on prognosis;
- 37 on prediction of treatment outcome;
- 19 on radiomics.

The included articles are listed in Supplementary Table S2.

Papers included have the following distribution over several decades:

- 18 papers in the years 1990–1999;
- 9 papers in the years 2000–2009;
- 41 papers in the years 2010–2020;
- 30 papers in the years 2020–2022.

The different types of contrast sequences used in the selected articles are represented by the following:

- Single post-contrast phase (12 papers);
- Dynamic contrast sequences (53 papers);
- Perfusion sequences (33 papers).

About DWI sequences:

- 62 articles do not have DWI sequences;
- 36 articles have the DWI sequences but only in 8 papers was there a comparison between DWI and CE-MRI.

### 3.2. Quality Assessment

**Figure 2:**
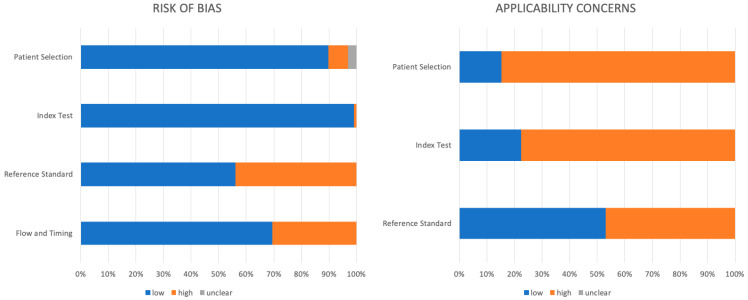
Results of QUADAS-2.

The results of QUADAS-2 are reported in Figure 2.

#### 3.2.1. Risk of Bias

For the patient selection, only seven papers had a high risk of bias, mainly because some patients were excluded from the analysis; the main reason for exclusion was that patients did not have surgery.

Only one paper was found at high risk of bias in the test index domain because the protocol was different among patients.

The situation is different regarding the reference standard and the flow and timing domains. Regarding the reference standard, 43 papers demonstrated a high risk of bias because of the lack of histopathological confirmation of radiological findings. In particular, in the group of articles about the prediction of treatment outcomes, a robust histopathological confirmation was missing. Regarding the workflow, the major problem was analyzing together patients undergoing different treatments in different time settings.

#### 3.2.2. Applicability Concerns

For the patient selection domain, 83 out of 98 papers have concerns about applicability. The main problem is the inhomogeneous population, causing the paper to consider together different stages of the disease, especially for older articles (the performance of MRI is different in various stages of the disease).

For the index test domain, the applicability concern was the absence of DWI-MR in the MRI protocol, which is now considered a fundamental part, in 62 articles; the second most frequent concern was the absence of a comparison between DWI-MR and CE-MRI (It is present in only eight papers).

Finally, for the standard reference domain, only 52 studies used histology as the reference test, significantly reducing the applicability of the analyzed studies.

## 4. Discussion

### 4.1. CE-MRI at Diagnosis

#### 4.1.1. Differentiation between Benign or Malignant Lesions

Regarding the differentiation of cervical cancer from benign cervical lesions, only one study compared the diagnostic accuracy of routine MRI (T1-weighted imaging -T1WI- and T2WI), DWI-MR, and CE-MRI using 3.0T equipment. Kuang et al. found that unenhanced MRI with combined DWI-MR and routine MRI at 3T can provide accurate information and may be preferable to CE-MRI [10].

#### 4.1.2. Origin of the Tumor (Endometrial vs. Endocervical)

When the histological diagnosis at the biopsy is an adenocarcinoma, differentiating between endometrial and cervical origin may be challenging. In this particular setting, we found four papers addressing this clinical problem. Two reported a good CE-MRI performance without specifically investigating the role of contrast in this setting [11,12]. Moreover, the other two papers were concordant for the utility of CE-sequences as cervical cancer was reported as hypervascular (both in qualitative [13] and quantitative [14] assessment) in comparison with endometrial cancer.

#### 4.1.3. Utility in Fertility-Sparing

A topic of common interest is the accurate staging and delineation of early-stage tumors to allow fertility-sparing (FS) treatment. The selection of the patients and the most appropriate FS surgical option depends on precise cancer staging. In this setting, MRI is suitable for defining the presence of eligibility criteria for conservative FS treatment.

We found four papers focused on the evaluation of CE-MRI in this setting.

Only one paper directly evaluated the role of CE-MRI. Akita et al. found that tumor margins appear more distinct on contrast-enhanced T1WI than on T2WI. This allowed better accuracy in detecting low-stage tumors [15]. However, in this study, DWI-MR was not used, limiting the significance of the results, considering that DWI-MR is a crucial sequence for cervical evaluation on MRI. On the other hand, Lakhman et al. investigated the role of MRI with DWI and CE sequences altogether, without considering the single contribution of different sequences; they found that its combination is a precise and reproducible tool for selecting patients for trachelectomy [16].

The other two studies that used dynamic contrast-enhanced magnetic resonance imaging (DCE-MRI) in this particular setting found a correlation between quantitative features of DCE-MRI with stage. Still, they did not comprehensively evaluate the MRI for selection for fertility-sparing treatment [17,18].

In the context of fertility-sparing treatment selection, we found a lack of robust evidence for the advantages of contrast enhancement despite it being usually suggested in this setting.

#### 4.1.4. Staging

We found 21 studies that investigated the use of CE-MRI in staging. Most studies compared the diagnostic accuracy of contrast and non-contrast sequences, using different types of sequences such as single-phase post-contrast acquisition [19,20,21,22,23,24,25,26,27,28], dynamic contrast enhancement [28,29,30,31,32,33,34,35,36,37,38], and perfusion sequences [39,40]. These studies reported different results. Some showed a decrease in diagnostic accuracy with the use of contrast agents [19,20,25,26,28,32,33] or did not reach statistical significance [10,23,24,27,31,36]. In contrast, others showed a slight improvement in diagnostic accuracy [15,17,21,22,29,30,34,37,39,40].

One study investigated different post-contrast sequences, testing the differences between isotropic and non-isotropic sequences; Yu et al. found a better signal-to-noise ratio in isotropic sequences but with a higher accuracy only for detecting vaginal invasion [38].

The bibliographic research shows that studies conducted to assess the diagnostic accuracy of contrast in MRI cases mostly date back to the 1990s and include small samples (below 100 patients) and often without comparison with DWI. More recent evidence investigated the usefulness of dynamic and perfusion sequences, trying to relate quantitative features with parametrial invasion. Papers described that DCE-MRI parameters are correlated with parametrial invasion in focally disrupted stromal ring IB–IIA cervical cancers [39,40]. However, these findings are no longer applicable in a clinical setting. On the other hand, it is reported that the post-contrast sequence may lead to an overestimation of the tumor extension caused by an intense accumulation of contrast around the tumor due to inflammation, and the use of DWI-MR allowed for the reduction of the number of false-positive results [19].

Based on the current literature, we can affirm that contrast-enhanced sequences have no clear advantage for cervical cancer staging.

### 4.2. Post-Treatment Evaluation

Over the last few decades, the role of DCE-MRI in post-treatment evaluation and how quantitative data extracted from DCE-MRI may help detect residual tumors after treatment have been a field of interest for scientific research.

#### 4.2.1. Detection of Tumor Recurrence after Fertility-Sparing Treatment

Early-stage cervical cancer can be conservatively treated by conization considering FS treatment. If the cone’s margins are positive, the clinicians want to assess the presence and extension of residual disease, and MRI can be helpful in this setting. However, the residual disease cannot always be detected by conventional MRI.

Only one article was found in this setting. Huang JW et al. evaluated whether quantitative data extracted from DCE-MRI may improve MRI diagnostic accuracy [41]. They enrolled 59 patients treated by conization, 35 of whom had histologically confirmed residual cervical cancer, invisible to MRI. Their results showed a significantly higher value of Ktrans and Ve in patients with residual cervical tumors, suggesting that perfusion may increase the identification of residual tumors undetectable on MRI [16]. However, other studies must confirm the results before this approach can be considered reliable.

#### 4.2.2. Detection after CCRT

Since 2015, the standard treatment for locally advanced cervical cancer (LACC) does not include a hysterectomy after CCRT. Therefore, detecting residual tumors after CCRT is extremely important in conservative management [42,43]. Unfortunately, MRI morphological sequences provide limited information regarding therapy response since tissue alterations caused by radiotherapy (i.e., edema, inflammation, and granulation tissue) are difficult to distinguish from a residual tumor; the addiction of DWI-MR can significantly improve diagnostic accuracy.

We found two original articles that investigated the role of CE-MRI in detecting residual disease after CCRT. First, Boss et al. conducted a pilot study, which included only 10 patients, and found that the early enhancement typical of the neoplasia was still present when a residual tumor was present. In contrast, the enhancement after treatment was lower in responders than at staging [44]. These results were in accordance with the more recent retrospective study by Jalaguier-Coudray et al. in 52 LACC who underwent neoadjuvant chemotherapy followed by hysterectomy. In addition, the study found that a perfusion time-signal intensity curve of cervical lesions that was steeper than the myometrial curve (i.e., an early and avid enhancement of irradiated tumor) was significantly associated with histologically confirmed residual tumors [45].

In conclusion, DCE-MRI is valuable in differentiating radiation-induced changes from residual disease or recurrence; however, we need to consider the scarcity of studies, which were retrospective and with a small number of patients. Therefore, other larger and prospective studies need to confirm this evidence.

### 4.3. Evaluation of Suspected Recurrence

Cervical cancer recurrence is defined as the regrowth of local tumors or the development of distant lesions after 6 months from the initial lesion [46]; almost 90% of recurrences are found within 5 years post-treatment [47]. The cervix, uterus, vagina, parametria, ovaries, bladder, or rectum are the most common sites of recurrences. Extra-pelvic organs may also be involved (para-aortic lymph nodes, lungs, liver, or bone) [48,49,50].

We found five articles that analyzed the use of CE-MRI in detecting recurrence [46,51,52,53,54]. However, three were published during the 1990s, and no comparison with DWI-MR was made as none of the articles had diffusion in the MR protocol. Apart from this bias, the findings of these old papers are conflicting: the paper with the most significant sample (69 patients) found that unenhanced images better detect recurrent tumors than enhanced ones, except for those few patients with adnexal or pelvic sidewall recurrences [52].

The other two studies (with 22 and 21 patients) found that dynamic CE-MRI better detects the recurrence [51,53].

When DWI-MR was included in the protocol, the performance of MRI in detecting recurrence improved. Two articles were published in 2015 on this topic. Mahajan et al. found that the best accuracy was reached by combining all different sequences in a multiparametric MRI, including experimental sequences such as BOLD hypoxia and spectroscopy; however, the single sequence that better characterized the recurrence was DWI-MR, with slightly better accuracy than DCE-MRI [54]. Lucas et al. directly compared the use of DWI-MR and DCE-MRI combined with T2WI; the best accuracy was found with the combination of T2WI with DWI-MR [46]. The authors suggest reserving the contrast injection only for ambiguous cases. In conclusion, the results are scarce and conflicting in this setting, and no clear advantage of contrast administration was found.

### 4.4. Prognosis

We found four articles that investigated if the perfusion pattern of the cervical tumor may help identify critical prognostic factors on staging exams [55,56,57,58].

Bai et al. investigated the correlation of perfusion of tumors with nodal involvement. The author described that tumor diameter and higher enhancement of the primary tumor were significantly higher in patients with nodal involvement [55].

Wang et al. investigated the role of DCE-MRI for lymphovascular space invasion detection and found that higher tumor contrast enhancement was significantly higher in those with invasion [56].

Two articles analyzed directly ambiguous lymph nodes in confirmed cervical cancer to identify metastatic ones. The findings were contradictory between the two studies: Kim et al. described that metastatic lymph nodes showed a significantly lower Ktrans [57], whereas, Zhang et al. stated that the perfusion parameters of metastatic lymph nodes were higher than those of non-metastatic lymph nodes [58].

Even if some correlation was found, these few studies did not provide strong evidence of CE-MRI’s role in better stratifying the prognosis of patients with cervical cancer.

### 4.5. Prediction of Treatment Response

CCRT is the treatment of choice for large cervical cancer LACC [6]. Pre- and mid-treatment MRIs are required to provide tailored treatment planning and RT dose adjustment to improve local tumor control and minimize the toxic effect of therapy.

Once initial treatment fails, further treatment options for cervical cancer are limited. Therefore, an accurate prediction of treatment response as early as possible is critical and may profoundly affect the prognosis of patients. Clinical prognostic factors (including stage, lymph node status, histology, and parametrial invasion) are currently well established to guide therapy selection [6]. Still, they do not always translate into successful treatment outcomes because of wide interindividual variability. Therefore, a reliable early marker of therapy response should be developed to provide a window of opportunity to modify and improve treatment strategies.

We found 37 papers on this topic. Many authors investigated the role of CE-MRI as a predictor of treatment response, mainly using quantitative perfusion values based on the correlation between tissue vascularization and treatment outcome.

Twenty studies investigated whether CE-MRI at the staging could provide information on predicting response to treatment [59,60,61,62,63,64,65,66,67,68,69,70,71,72,73,74,75,76,77,78]. The perfusion technique is the preferred contrast modality in this field. All those studies found a difference in perfusion characteristics between responders and non-responders. In particular, the poorly perfused-hypoxic tumor is linked to increased tumor aggressiveness, increased risk of metastasis, and treatment failure with less local control; tumors that showed a lower enhancement (poorly perfused) had a worse response to therapy as well as a lower survival rate. However, those studies did not identify a precise and reproducible value of those parameters, and this evidence’s impact on clinical practice is unclear.

In 17 studies, a longitudinal approach was used, using more than one MRI to perform an early response prediction [79,80,81,82,83,84,85,86,87,88,89,90,91,92,93,94,95].

Fifteen studies involved patients with LACC who had undergone CCRT using MRI performed before and during the first weeks of treatment (2–2.5 weeks from the beginning). The other two studies, on the other hand, included patients undergoing neoadjuvant chemotherapy (NACT) and MRI examinations performed before, during, and after therapies. High tumor enhancement on pre-treatment MRI and increased perfusion seen during the first few weeks of RT were correlated with reduced hypoxia levels in the tumor tissue, which was associated with increased radiosensitivity, tumor regression, and locoregional control. Conversely, Hawighorst et al. argued that high tumor perfusion was supported by high angiogenesis, an indicator of tumor aggressiveness, and, therefore, poor prognosis [85].

Again, even if many papers found a significant correlation between perfusion characteristics of the tumor and response to treatment, findings could not build a robust univocal model to identify non-responders to treatment early; therefore, none of those findings were translated into clinical practice.

### 4.6. Radiomics

Radiomics represents an emerging field of interest in the scientific landscape that provides quantitative microscopic and mesoscopic tissue characteristics from clinical images. Combining radiomics data (such as intensity features reflecting the signal intensity, size- and shape-related features, and texture features, measured the relationship between each tumor voxel and its environments in order to detect intra-tumor heterogeneity) with other information (histology, molecular data, clinical information, etc.), it is possible to build new models to predict different targets: diagnosis, histotype, therapeutic response, prognosis, and survival.

Nineteen studies used CE-MRI-based radiomics models in cervical cancer [96,97,98,99,100,101,102,103,104,105,106,107,108,109,110,111,112,113,114]. Different outcomes were investigated; some of the papers investigated more than one outcome; the most frequently tested radiomics results were the prediction of complete response, nodal involvement, progression-free survival, and overall survival and vaginal involvement.

All of them showed a better performance in radiomics or combined clinico-radiomics models than the clinical ones, suggesting that radiomics might be used as a prognostic biomarker and helpful in tailoring therapeutic management.

Although radiomics is gaining a growing interest and offers a wide range of possible applications, it still needs more standardization, validation, biological correlation, and interpretation; more robust studies are required before introducing radiomics tools in clinical practice.

## 5. Conclusions

From our literature review, it emerges that many papers about the use of contrast in cervical cancer are old and outdated, with a high proportion of them having a high probability of bias and concerns about applicability, especially those regarding staging and detection of tumor recurrence. A significant part of the most recent literature focuses on the prediction of outcomes after chemo-radiotherapy.

We did not find strong evidence suggesting that CE-MRI is helpful in the clinical setting for cervical cancer staging and detection of tumor recurrence. So, from the results of our research, the use of contrast may be avoided. There is evidence that contrast-enhanced sequences do not add valuable information to DWI-MR.

In detecting residual tumors after CCRT, the DCE-MRI parameters may help, but the evidence is not strong enough to suggest its use in clinical practice. Other prospective studies focusing on the role of DCE-MRI parameters in direct comparison with DWI are necessary to investigate the real significance of these results.

There is growing evidence that perfusion parameters and perfusion-derived radiomics models might have a role as prognostic and predictive biomarkers. Still, the lack of standardization and validation limits its use only in research settings. More extensive multicentric studies, with robust external validation, focusing on prognostic information derived from perfusion parameters are needed for future research to introduce it in daily clinical practice.

## Data Availability

The research criteria are fully described and papers were found in publicly available search engines.

## Supplementary Materials

The following supporting information can be downloaded at: https://www.mdpi.com/article/10.3390/life13061368/s1; Table S1: PRISMA checklist; Table S2: List of included papers.
