# Supplementary material for: The Utility of Contrast-Enhanced Magnetic Resonance Imaging in Uterine Cervical Cancer: A Systematic Review
Source: Life (Basel). 2023 Jun 12;13(6):1368. doi: 10.3390/life13061368 (PMC10303560; doi:10.3390/life13061368)
Supplement: Supplementary file 1 [file life-13-01368-s001.zip › life-2356714-supplementary.pdf]

PRISMA 2020 Checklist

Section and Topic	Item #	Checklist item	Location where item is reported
**TITLE**			
Title	1	Identify the report as a systematic review.	Page 1
**ABSTRACT**			
Abstract	2	See the PRISMA 2020 for Abstracts checklist.	Page 1
**INTRODUCTION**			
Rationale	3	Describe the rationale for the review in the context of existing knowledge.	Page 2
Objectives	4	Provide an explicit statement of the objective(s) or question(s) the review addresses.	Page 2
**METHODS**			
Eligibility criteria	5	Specify the inclusion and exclusion criteria for the review and how studies were grouped for the syntheses.	Page 3
Information sources	6	Specify all databases, registers, websites, organisations, reference lists and other sources searched or consulted to identify studies. Specify the date when each source was last searched or consulted.	Page 3
Search strategy	7	Present the full search strategies for all databases, registers and websites, including any filters and limits used.	Page 3
Selection process	8	Specify the methods used to decide whether a study met the inclusion criteria of the review, including how many reviewers screened each record and each report retrieved, whether they worked independently, and if applicable, details of automation tools used in the process.	Page 3
Data collection process	9	Specify the methods used to collect data from reports, including how many reviewers collected data from each report, whether they worked independently, any processes for obtaining or confirming data from study investigators, and if applicable, details of automation tools used in the process.	Page 3
Data items	10a	List and define all outcomes for which data were sought. Specify whether all results that were compatible with each outcome domain in each study were sought (e.g. for all measures, time points, analyses), and if not, the methods used to decide which results to collect.	Page 4
	10b	List and define all other variables for which data were sought (e.g. participant and intervention characteristics, funding sources). Describe any assumptions made about any missing or unclear information.	Page 4
Study risk of bias assessment	11	Specify the methods used to assess risk of bias in the included studies, including details of the tool(s) used, how many reviewers assessed each study and whether they worked independently, and if applicable, details of automation tools used in the process.	Page 3
Effect measures	12	Specify for each outcome the effect measure(s) (e.g. risk ratio, mean difference) used in the synthesis or presentation of results.	Pages 3-4
Synthesis methods	13a	Describe the processes used to decide which studies were eligible for each synthesis (e.g. tabulating the study intervention characteristics and comparing against the planned groups for each synthesis (item #5)).	Pages 3-4
	13b	Describe any methods required to prepare the data for presentation or synthesis, such as handling of missing summary statistics, or data conversions.	Pages 3-4
	13c	Describe any methods used to tabulate or visually display results of individual studies and syntheses.	Pages 3-4
	13d	Describe any methods used to synthesize results and provide a rationale for the choice(s). If meta-analysis was performed, describe the model(s), method(s) to identify the presence and extent of statistical heterogeneity, and software package(s) used.	Pages 3-4
	13e	Describe any methods used to explore possible causes of heterogeneity among study results (e.g. subgroup analysis, meta-regression).	Pages 3-4
	13f	Describe any sensitivity analyses conducted to assess robustness of the synthesized results.	Pages 3-4
Reporting bias assessment	14	Describe any methods used to assess risk of bias due to missing results in a synthesis (arising from reporting biases).	Pages 3-4
Certainty assessment	15	Describe any methods used to assess certainty (or confidence) in the body of evidence for an outcome.	Pages 3-4

PRISMA 2020 Checklist

Section and Topic	Item #	Checklist item	Location where item is reported
**RESULTS**			
Study selection	16a	Describe the results of the search and selection process, from the number of records identified in the search to the number of studies included in the review, ideally using a flow diagram.	Page 4
	16b	Cite studies that might appear to meet the inclusion criteria, but which were excluded, and explain why they were excluded.	Page 4
Study characteristics	17	Cite each included study and present its characteristics.	Table S2
Risk of bias in studies	18	Present assessments of risk of bias for each included study.	Page 5
Results of individual studies	19	For all outcomes, present, for each study: (a) summary statistics for each group (where appropriate) and (b) an effect estimate and its precision (e.g. confidence/credible interval), ideally using structured tables or plots.	Pages 6-9
Results of syntheses	20a	For each synthesis, briefly summarise the characteristics and risk of bias among contributing studies.	Pages 6-9
	20b	Present results of all statistical syntheses conducted. If meta-analysis was done, present for each the summary estimate and its precision (e.g. confidence/credible interval) and measures of statistical heterogeneity. If comparing groups, describe the direction of the effect.	Pages 6-9
	20c	Present results of all investigations of possible causes of heterogeneity among study results.	Pages 6-9
	20d	Present results of all sensitivity analyses conducted to assess the robustness of the synthesized results.	Pages 6-9
Reporting biases	21	Present assessments of risk of bias due to missing results (arising from reporting biases) for each synthesis assessed.	Page 5
Certainty of evidence	22	Present assessments of certainty (or confidence) in the body of evidence for each outcome assessed.	
**DISCUSSION**			
Discussion	23a	Provide a general interpretation of the results in the context of other evidence.	Pages 6-10
	23b	Discuss any limitations of the evidence included in the review.	Pages 6-10
	23c	Discuss any limitations of the review processes used.	Pages 6-10
	23d	Discuss implications of the results for practice, policy, and future research.	Page 10
**OTHER INFORMATION**			
Registration and protocol	24a	Provide registration information for the review, including register name and registration number, or state that the review was not registered.	Page 3
	24b	Indicate where the review protocol can be accessed, or state that a protocol was not prepared.	Page 3
	24c	Describe and explain any amendments to information provided at registration or in the protocol.	Page 3
Support	25	Describe sources of financial or non-financial support for the review, and the role of the funders or sponsors in the review.	Page 10
Competing interests	26	Declare any competing interests of review authors.	Page 10
Availability of data, code and other materials	27	Report which of the following are publicly available and where they can be found: template data collection forms; data extracted from included studies; data used for all analyses; analytic code; any other materials used in the review.	Page 10

From: Page MJ, McKenzie JE, Bossuyt PM, Boutron I, Hoffmann TC, Mulrow CD, et al. The PRISMA 2020 statement: an updated guideline for reporting systematic reviews. BMJ 2021;372:n71. doi: 10.1136/bmj.n71

For more information, visit: http://www.prisma-statement.org/

Authors	Date	Title	DOI
Hricak et al	1991	Carcinoma of the uterus: use of gadopentetate dimeglumine in MR imaging	10.1148/radiology.181.1.1887062
Thurnher et al	1992	MR imaging of pelvic masses in women: contrast-enhanced vs unenhanced images	10.2214/ajr.159.6.1442393
Yamashita et al	1992	Carcinoma of the cervix: dynamic MR imaging	10.1148/radiology.182.3.1535875
Hanabayashi et al	1993	Enhanced magnetic resonance imaging evaluation for spread of cervical carcinoma	10.1016/0020-7292(93)90519-3
Sironi et al	1993	Carcinoma of the cervix: value of plain and gadolinium-enhanced MR imaging in assessing degree of invasiveness	10.1148/radiology.188.3.8351350
Hricak et al	1993	Irradiation of the cervix uteri: value of unenhanced and contrast-enhanced MR imaging	10.1148/radiology.189.2.8210364
Hawighorst et al	1996	Cervical carcinoma: comparison of standard and pharmacokinetic MR imaging	10.1148/radiology.201.2.8888254
Seki et al	1996	Stromal invasion by carcinoma of the cervix: assessment with dynamic MR imaging	10.2214/ajr.168.6.9168730
Yamashita et al	1996	Dynamic MR imaging of recurrent postoperative cervical cancer	10.1002/jmri.1880060130
Hawighorst	1996	Pelvic lesions in patients with treated cervical carcinoma: efficacy of pharmacokinetic analysis of dynamic MR images in distinguishing recurrent tumors from benign conditions	10.2214/ajr.166.2.8553955
Mayr et al	1996	Tumor perfusion studies using fast magnetic resonance imaging technique in advanced cervical cancer: a new noninvasive predictive assay	10.1016/s0360-3016(97)85090-0
Tsuda et al	1997	MR imaging of cervical carcinoma: comparison among T2-weighted, dynamic, and postcontrast T1-weighted images with histopathological correlation	10.1007/s002619900151
Abe et al	1998	High-resolution turbo spin-echo MR imaging with contrastenhanced dynamic scanning and T2-weighting	10.1080/02841859809172203
Postema et al	1998	Fast dynamic contrast-enhanced colour-coded MRI in uterine cervix carcinoma: useful for tumour staging?	10.1016/s0009-9260(98)80314-0
Scheidler et al	1998	Parametrial invasion in cervical carcinoma: evaluation of detection at MR imaging with fat suppression	10.1148/radiology.206.1.9423661
Van Vierzen et al	1998	Fast dynamic contrast enhanced MR imaging of cervical carcinoma	10.1016/s0009-9260(98)80098-6
Postema et al	1999	Cervical carcinoma: can dynamic contrast-enhanced MR imaging help predict tumor aggressiveness?	10.1148/radiology.210.1.r99ja16217
Gong et al	1999	Contrast enhanced dynamic MRI of cervical carcinoma during radiotherapy: early prediction of tumour regression rate	10.1259/bjr.72.864.10703475
Mayr et al	2000	Pixel analysis of MR perfusion imaging in predicting radiation therapy outcome in cervical cancer	10.1002/1522-2586(200012)12:6<1027::aid-jmri31>3.0.co;2-5
Boss et al	2001	Post-radiotherapy contrast enhancement changes in fast dynamic MRI of cervical carcinoma	10.1002/jmri.1084
Sironi et al	2002	Clinical stage I carcinoma of the uterine cervix value of preoperative magnetic resonance imaging in assessing parametrial invasion	10.1177/030089160208800410
Loncaster et al	2002	Prediction of radiotherapy outcome using dynamic contrast enhanced MRI of carcinoma of the cervix	10.1016/s0360-3016(02)02972-3
Dedieu et al	2008	Capillary permeability and extracellular volume fraction in uterine cervical cancer as patient outcome predictors: measurements by using dynamic MRI spin-lattice relaxometry	10.1002/jmri.21324
Zahra et al	2009	Semiquantitative and quantitative dynamic contrast-enhanced magnetic resonance imaging measurements predict radiation response in cervix cancer	10.1016/j.ijrobp.2008.08.023

Semple et al	2009	A combined pharmacokinetic and radiologic assessment of dynamic contrast-enhanced magnetic resonance imaging predicts response to chemoradiation in locally advanced cervical cancer	10.1016/j.ijrobp.2009.04.069
Yuh et al	2009	Predicting control of primary tumor and survival by DCE MRI during early therapy in cervical cancer	10.1097/RLI.0b013e3181a64ce9
Mayr et al	2009	Synergistic effects of hemoglobin and tumor perfusion on tumor control and survival in cervical cancer	10.1016/j.ijrobp.2008.09.050
Mannelli et al	2010	Evaluation of nonenhancing tumor fraction assessed by dynamic contrast-enhanced MRI subtraction as a predictor of decrease in tumor volume in response to chemoradiotherapy in advanced cervical cancer	10.2214/AJR.09.3437
Mayr et al	2010	Longitudinal changes in tumor perfusion pattern during the radiation therapy course and its clinical impact in cervical cancer	10.1016/j.ijrobp.2009.04.084
Mayr et al	2010	Ultra-early predictive assay for treatment failure using functional magnetic resonance imaging and clinical prognostic parameters in cervical cancer	10.1002/cncr.24822
Vargas et al	2011	The value of MR imaging when the site of uterine cancer origin is uncertain	10.1148/radiol.10101147
Lin et al	2011	Role of magnetic resonance imaging and apparent diffusion coefficient at 3T in distinguishing between adenocarcinoma of the uterine cervix and endometrium	
Akita et al	2011	Comparison of T2-weighted and contrast-enhanced T1-weighted MR imaging at 1.5 T for assessing the local extent of cervical carcinoma	10.1007/s00330-011-2122-6
Andersen et al	2011	Dynamic contrast-enhanced MRI of cervical cancers: temporal percentile screening of contrast enhancement identifies parameters for prediction of chemoradioresistance	10.1016/j.ijrobp.2011.05.050
Huang et al	2012	Characterizing at-Risk Voxels by Using Perfusion Magnetic Resonance Imaging for Cervical Cancer during Radiotherapy	10.4172/1948-5956.1000151
Mayr et al	2012	Characterizing tumor heterogeneity with functional imaging and quantifying high-risk tumor volume for early prediction of treatment outcome: cervical cancer as a model	10.1016/j.ijrobp.2011.08.011
Kim et al	2012	Dynamic contrast-enhanced 3-T MR imaging in cervical cancer before and after concurrent chemoradiotherapy	10.1007/s00330-012-2504-4
He et al	2013	MRI is highly specific in determining primary cervical versus endometrial cancer when biopsy results are inconclusive	10.1016/j.crad.2013.05.095
Lin et al	2013	Evaluation of international federation of gynecology and obstetrics stage IB cervical cancer: comparison of diffusion-weighted and dynamic contrast-enhanced magnetic resonance imaging at 3.0 T	10.1097/RCT.0b013e3182990a65
Andersen et al	2013	Pharmacokinetic parameters derived from dynamic contrast enhanced MRI of cervical cancers predict chemoradiotherapy outcome	10.1016/j.radonc.2012.11.007
Hompland et al	2014	Peritumoral interstitial fluid flow velocity predicts survival in cervical carcinoma	10.1016/j.radonc.2014.09.011
Park et al	2014	Assessment of early response to concurrent chemoradiotherapy in cervical cancer: value of diffusion-weighted and dynamic contrast-enhanced MR imaging	10.1016/j.mri.2014.05.009
Himoto et al	2014	Assessment of the early predictive power of quantitative magnetic resonance imaging parameters during neoadjuvant chemotherapy for uterine cervical cancer	10.1097/IGC.0000000000000124
Torheim et al	2014	Classification of dynamic contrast enhanced MR images of cervical cancers using texture analysis and support vector machines	10.1109/TMI.2014.2321024
Kuang et al	2015	Diagnostic accuracy of diffusion-weighted MRI for differentiation of cervical cancer and benign cervical lesions at 3.0T: Comparison with routine MRI and dynamic contrast-enhanced MRI	10.1002/jmri.24894
Yu et al	2015	Comparison of contrast-enhanced isotropic 3D-GRE-T1WI sequence versus conventional non-isotropic sequence on preoperative staging of cervical cancer	10.1371/journal.pone.0122053

Lucas et al	2015	Added value of diffusion-weighted MRI in detection of cervical cancer recurrence: comparison with morphologic and dynamic contrast-enhanced MRI sequences	10.5152/dir.2015.14427
Mahajan et al	2015	Role of 3T multiparametric-MRI with BOLD hypoxia imaging for diagnosis and post therapy response evaluation of postoperative recurrent cervical cancers	10.1016/j.ejro.2015.11.003
Lund et al	2015	Short-term pretreatment DCE-MRI in prediction of outcome in locally advanced cervical cancer	10.1016/j.radonc.2015.05.001
Fridsten et al	2016	Preoperative MR staging of cervical carcinoma: are oblique and contrast-enhanced sequences necessary?	10.1177/2058460116679460
Torheim et al	2016	Cluster analysis of dynamic contrast enhanced MRI reveals tumor subregions related to locoregional relapse for cervical cancer patients	10.1080/0284186X.2016.1189091
Onal et al	2016	Treatment response evaluation using the mean apparent diffusion coefficient in cervical cancer patients treated with definitive chemoradiotherapy	10.1002/jmri.25215
Jalaguier-Coudray et al	2017	Value of Dynamic Contrast-enhanced and Diffusion-weighted MR Imaging in the Detection of Pathologic Complete Response in Cervical Cancer after Neoadjuvant Therapy: A Retrospective Observational Study	10.1148/radiol.2017161299
Lund et al	2017	Pretreatment late-phase DCE-MRI predicts outcome in locally advanced cervix cancer	10.1080/0284186X.2017.1294762
Dickie et al	2017	The prognostic value of dynamic contrast-enhanced MRI contrast agent transfer constant Ktrans in cervical cancer is explained by plasma flow rather than vessel permeability	10.1038/bjc.2017.121
Huang et al	2018	Making the invisible visible: improving detectability of MRI-invisible residual cervical cancer after conisation by DCE-MRI	10.1016/j.crad.2018.10.013
Simonsen et al	2018	DCE-MRI-Derived Measures of Tumor Hypoxia and Interstitial Fluid Pressure Predict Outcomes in Cervical Carcinoma	10.1016/j.ijrobp.2018.04.035
Zhang et al	2018	Dynamic Contrast-Enhanced Magnetic Resonance Imaging of Advanced Cervical Carcinoma: The Advantage of Perfusion Parameters From the Peripheral Region in Predicting the Early Response to Radiotherapy	10.1097/IGC.0000000000001308
Yang et al	2018	Multi-parametric MRI in cervical cancer: early prediction of response to concurrent chemoradiotherapy in combination with clinical prognostic factors	10.1007/s00330-017-4989-3
Bowen et al	2018	Tumor radiomic heterogeneity: Multiparametric functional imaging to characterize variability and predict response following cervical cancer radiation therapy	10.1002/jmri.25874
Bakai et al	2019	Radiation research to determine local tumor invasion in patients with cervical cancer	10.32471/exp-oncology.2312-8852.vol-41-no-1.12596
Song et al	2019	Value of diffusion-weighted and dynamic contrast-enhanced MR in predicting parametrial invasion in cervical stromal ring focally disrupted stage IB-IIA cervical cancers	10.1007/s00261-019-02107-y
Feng et al	2019	Combined dynamic DCE-MRI and diffusion-weighted imaging to evaluate the effect of neoadjuvant chemotherapy in cervical cancer	10.1177/0300891619886656
Li et al	2019	MR-Based Radiomics Nomogram of Cervical Cancer in Prediction of the Lymph-Vascular Space Invasion preoperatively	10.1002/jmri.26531
Lu et al	2019	On the potential use of dynamic contrast-enhanced (DCE) MRI parameters as radiomic features of cervical cancer	10.1002/mp.13821
Kan et al	2019	Radiomic signature as a predictive factor for lymph node metastasis in early-stage cervical cancer	10.1002/jmri.26209
Wu et al	2019	Radiomics Analysis of Multiparametric MRI Evaluates the Pathological Features of Cervical Squamous Cell Carcinoma	10.1002/jmri.26301
Lin et al	2020	Differentiation of endometrial adenocarcinoma from adenocarcinoma of cervix using kinetic parameters derived from DCE-MRI	10.1016/j.ejrad.2020.109190

Li et al	2020	Diagnosis of Cervical Cancer With Parametrial Invasion on Whole-Tumor Dynamic Contrast-Enhanced Magnetic Resonance Imaging Combined With Whole-Lesion Texture Analysis Based on T2- Weighted Images	10.3389/fbioe.2020.00590
Bai et al	2020	Quantitative kinetic parameters of primary tumor can be used to predict pelvic lymph node metastasis in early-stage cervical cancer	10.1007/s00261-020-02762-6
Kim et al	2020	Assessment of pelvic lymph node metastasis in FIGO IB and IIA cervical cancer using quantitative dynamic contrast-enhanced MRI parameters	10.5152/dir.2020.19365
Zhang et al	2020	The Role of Multiparametric Magnetic Resonance Imaging in the Study of Primary Tumor and Pelvic Lymph Node Metastasis in Stage IB1-IIA1 Cervical Cancer	10.1097/RCT.0000000000001084
Liu et al	2020	DCE-MRI Quantitative Parameters as Predictors of Treatment Response in Patients With Locally Advanced Cervical Squamous Cell Carcinoma Underwent CCRT	10.3389/fonc.2020.585738
Lund et al	2020	DCE-MRI of locally-advanced carcinoma of the uterine cervix: Tofts analysis versus non-model-based analyses	10.1186/s13014-020-01526-2
Zheng et al	2020	Prediction of early response to concurrent chemoradiotherapy in cervical cancer: Value of multi-parameter MRI combined with clinical prognostic factors	10.1016/j.mri.2020.06.014
Zhang et al	2020	Combined dynamic contrast-enhanced magnetic resonance imaging and diffusion-weighted imaging to predict neoadjuvant chemotherapy effect in FIGO stage IB2-IIA2 cervical cancers	10.1007/s11547-020-01214-x
Fang et al	2020	Association of MRI-derived radiomic biomarker with disease-free survival in patients with early-stage cervical cancer	10.7150/thno.37429
Park et al	2020	Magnetic resonance imaging features of tumor and lymph node to predict clinical outcome in node-positive cervical cancer: a retrospective analysis	10.1186/s13014-020-01502-w
Wang et al	2020	MRI texture features differentiate clinicopathological characteristics of cervical carcinoma	10.1007/s00330-020-06913-7
Hou et al	2020	Radiomics Analysis of Multiparametric MRI for the Preoperative Prediction of Lymph Node Metastasis in Cervical Cancer	10.3389/fonc.2020.01393
Matsumoto et al	2021	Diagnostic Accuracy of Magnetic Resonance Imaging for International Federation of Gynecology and Obstetrics 2018 IB to IIB Cervical Cancer Staging: Comparison Among Magnetic Resonance Sequences and Pathologies	10.1097/RCT.0000000000001210
Gaustad et al	2021	Assessment of Hypoxic Tissue Fraction and Prediction of Survival in Cervical Carcinoma by Dynamic Contrast-Enhanced MRI	10.3389/fonc.2021.668916
Qin et al	2021	Combined dynamic contrast enhanced MRI parameter with clinical factors predict the survival of concurrent chemo-radiotherapy in patients with 2018 FIGO IIICr stage cervical cancer	10.1016/j.ejrad.2021.109787
Zhang et al	2021	Added-value of dynamic contrast-enhanced MRI on prediction of tumor recurrence in locally advanced cervical cancer treated with chemoradiotherapy	10.1007/s00330-021-08279-w
Lu et al	2021	The Role of Dynamic Contrast-Enhanced Magnetic Resonance Imaging in Predicting Treatment Response for Cervical Cancer Treated with Concurrent Chemoradiotherapy	10.2147/CMAR.S314289
Jiang et al	2021	MRI Based Radiomics Approach With Deep Learning for Prediction of Vessel Invasion in Early-Stage Cervical Cancer	10.1109/TCBB.2019.2963867
Bhardwaj et al	2022	Added-Value of Diffusion-Weighted Imaging (DWI) and Dynamic Contrast-Enhanced (DCE-MRI) Magnetic Resonance Imaging in the Preoperative Assessment of Cervical Cancer	10.1007/s13224-021-01488-9
Bai et al	2022	ADC and kinetic parameter of primary tumor: Surrogate imaging markers for fertility-sparing vaginal radical trachelectomy in patients with stage IB cervical cancer	10.1016/j.ejrad.2022.110467
Wang et al	2022	Preliminary MRI Study of Extracellular Volume Fraction for Identification of Lymphovascular Space Invasion of Cervical Cancer	10.1002/jmri.28423
Hu et al	2022	Added value of radiomics analysis in MRI invisible early-stage cervical cancers	10.1259/bjr.20210986

Zhao et al	2022	Classifying early stages of cervical cancer with MRI-based radiomics	10.1016/j.mri.2022.03.002
Kim et al	2022	Magnetic resonance imaging-based texture analysis for the prediction of postoperative clinical outcome in uterine cervical cancer	10.1007/s00261-021-03288-1
Jiang et al	2022	MRI radiomics combined with clinicopathologic features to predict disease-free survival in patients with early-stage cervical cancer	10.1259/bjr.20211229
Zhang et al	2022	MRI-based radiomics value for predicting the survival of patients with locally advanced cervical squamous cell cancer treated with concurrent chemoradiotherapy	10.1186/s40644-022-00474-2
Huang et al	2022	Multi-Parametric Magnetic Resonance Imaging-Based Radiomics Analysis of Cervical Cancer for Preoperative Prediction of Lymphovascular Space Invasion	10.3389/fonc.2021.663370
Zhou et al	2022	Multiparametric magnetic resonance imaging-derived radiomics for the prediction of disease-free survival in early-stage squamous cervical cancer	10.1007/s00330-021-08326-6
Wang et al	2022	Multiparametric MRI-based radiomics analysis: differentiation of subtypes of cervical cancer in the early stage	10.1177/02841851211014188

Authors	Date	Title	DOI
Lakman et al	2013	Stage IB1 Cervical Cancer: Role of Preoperative MR Imaging in Selection of Patients for Fertility-Sparing Radical Trachelectomy	https://doi.org/10.1148/radiol.13121746
